# Excerpta

**Published:** 1881-04

**Authors:** 


### EXCERPTA.

### THE TREATMENT OF BURNS.

The London *Medical Record* gives the treatment pursued by a writer, who, as a physician in chief to a large iron mining and smelting company in Styria, has cases of burns very frequently presented for treatment, and has found the following method most successful. The wound is first cleaned (without opening the blisters), then disinfected with a two per cent. solution of carbolic acid, and covered with a thick furniture varnish, prepared from linseed oil and litharge, in which five per cent. of salicylic acid has been dissolved by warming. The varnish is allowed to dry and another coat applied; over this a layer of Bruns' cotton is placed, about two thirds centimeter in thickness. The wound seldom suppurates, generally healing under the varnish, which is finally removed as a dry pellicle, no change of dressing having been necessary. Should, however, suppuration be indicated by the setting in of fever, or by the occurrence of painfulness, the dressing is removed from this locality; if the spot be not over five centimeters in diameter, nothing further is done than to strew the moist surface with dry powdered salicylic acid; if larger, an opening is cut in the dressing, the salicylic acid is applied as before, and then covered with a fresh layer of cotton. The scars become, by this method, entirely smooth, white, and are not hypertrophied. *Med. and Surg. Reporter.*

### QUINIA FROM COAL TAR.

The New York Commercial Bulletin notices a report current in that city that a Liberty-street firm had applied for a patent for a process to manufacture quinia from coal tar. It is stated that the firm has been interested with a chemist to accomplish this result for several years, and that the efforts have been crowned with success. In the "Chemical Notes" published in this Journal during the past year the readers have been kept informed of the results obtained by Skraup, Koenigs, Hesse, and others in the endeavors to determine the exact composition of the cinchona alkaloids, which when once known will doubtless lead to their synthetical production. Whether such a result is so near being accomplished as the rumor mentioned above indicates, remains to be seen; but of the importance of the synthetical production of quinia there can be no doubt, when it is remembered that in the year of 1879, two million dollars' worth of cinchona bark was imported, most of which has doubtless been used in the manufacture of this indispensable alkaloid.—*J. M. M., in Am. Jour. of Pharmacy.*

### A GERMAN VIEW OF THE QUALIFICATIONS OF A PHYSICIAN.

Verily the vocation of the earnest physician has always been difficult, and it will be more difficult still if he devotes his time chiefly to social and humane pursuits. By no means does it suffice to study the structure of the human body and its functions in health and disease, to practice in surgical technology and the pharmaceutical art of prescribing. The physician must penetrate the thousand fold relations of civilized society, in its highest and lowest spheres. He must be conversant with all the intellectual processes and their condition of development, and must have a clear view of the results and limits of all natural science. To him belongs the " nihil humani a me alienum " in its fullest comprehension. He must possess not only the eye of an acute searcher, but all his mental powers must be educated in an efficient and harmonious manner, not less in the direction of sensibility than of intellect. This can not be effected by many years of university study. His physiological schooling must begin much earlier and must continue much later. On earth there is no object of study greater and more beautiful than man. He is the most difficult and the most sublime subject for thought and work. You must bring to your task a clear eye and sharp ears ; acuteness of observation and patience for infinite study ; an uncloudy brain and an iron will strengthened in difficulty and embarrassment, but a warm, loving heart which takes cognizance of all sorrow and sympathizes with it ; religious conviction and moral stamina which resist the seductions of sensuality, money and honors. Besides, you must have a respectable exterior ; you must be polished in conversation ; dexterous with the hands ; possessed of health of body and soul. All this you must be if you would avoid misfortune and failure as a physician. You must carry the camel's burthen of knowledge and preserve the freshness of the poet. You must weigh all the tricks of charlatanism and in the midst of temptation remain an honest man. Remember that on your calling depends everything."

[After reading the above once, read it again, and then think about it. Almost every point there presented is as applicable to the dentist as to the general physician.

One of the great disabilities that attach to the dental profession is a want of appreciation of the qualities and attainments here referred to.—ED. REG.]—*Albert S. Tadler, M. D., in Dental Register.*

### THE ACTIVE PRINCIPLE OF HYDRASTIS.

At a recent meeting of the Pharmaceutical Society of Philadelphia, Dr. L. Wolff made some remarks upon an improved method of obtaining the alkaloids of *hydrastis canadensis*, which he thought worthy of notice by those making them. It consists in exhausting the root of the fatty oils by percolating with gasolin before proceeding to prepare the alkaloids; the advantage derived by this proceedure is quite considerable; the purification and crystallization of the hydrastia is effected with greatly less trouble. An inquiry was made whether hydrastia had any remedial powers other than those of the tonic effects of the berberina associated with it, which was answered by a positive statement that the astringent effects and the control of unnatural discharges were due to the hydrastia, while berberina had a remedial power as a tonic and cholagogue of its own.—*Medical and Surgical Reporter.*

### TRACHEOTOMY WITHOUT TUBES.

Dr. Alfred C. Post, of New York, writes, in the *Annals of Anatomy and Surgery*:—

My personal experience in tracheotomy without tubes, according to the method suggested by Dr. H. A. Martin, of Boston, is confined to two cases. In both of these cases the operations were performed as preliminary measures to facilitate the continued administration of anæsthetics, and to guard against the entrance of blood into the bronchial tubes in protracted and bloody operations involving the nasal and buccal cavities. In each instance the practical result was, in the highest degree, satisfactory, as compared with the operation as ordinarily performed with the introduction of a tracheal tube. The opening into the trachea was larger and more direct, not liable to be clogged with mucus, and unattended with the irritation which is often occasioned by the presence of the tube. The fauces being distended with a large sponge, having a strong cord attached to it, and hanging out of the mouth, scarcely any blood found its way into the trachea. The small quantity which escaped into the respiratory tube was readily absorbed by a small sponge attached to a handle. The apparatus for administering the anæsthetic was applied over the tracheal opening as often and as long as was found to be necessary, and without interfering in any way with the progress of the operation. As far as my limited experience in these two cases will warrant an opinion, I am disposed to recommend, in the strongest terms, the operations without the introduction of a tube.

When tracheotomy is performed for the relief of obstruction to the introduction of air through the larynx, and where the opening is required to be maintained for a considerable time, it is possible that practical difficulties may be encountered in maintaining the opening without the tube. But I am strongly inclined to the opinion that such difficulties may be obviated by carefully attaching the skin to the tracheal opening by a number of fine sutures.

I am fully persuaded that the use of the tracheal tube is a source of irritation, and that it is very desirable to dispense with it if the object of the operation can be accomplished without it.—*Medical and Surgical Reporter.*

Street Scene in Philadelphia.—A crowd attracts Dr. Chapman's attention; he draws up and, recognizing one of his pupils, hails him and inquires: "What's the matter?" "Ah, doctor, I am glad to see you. A young lady has fallen and sprained her ankle." "All right, set it;" replied the doctor, "but don't get above your business."—*Ohio Medical Reporter.*

Chloral Hydrate for Toothache.—Dr. Sporer has found that chloral hydrate applied to a decayed tooth will often give instant relief. He puts three or four crystals (about 5 centigrammes) on a piece of cotton wool, and applies it direct.—*Eclectic Med. Journal.*

### DEATH FROM AN AMALGAM PLUG.

The following case of mal-practice by an itinerant dentist, and the subsequent false diagnosis by physicians, should be a warning both to the community in employing quacks for the sake of getting cheap dentistry, and to the medical profession in ignoring the specific province of the dental profession:

Dr.(?)—— calling on Mr. R. for something to do in the way of dentistry, was given the filling of an upper molar. It was filled with amalgam, the remark being made by the dentist at the time that it had taken more "paste" than any tooth he had ever filled.

Two months afterward a gentleman called on me saying Mr. R. was suffering terribly with pain in his face, and giving the above facts, asked my advice. I said I could say nothing positive without seeing the patient, but that probably the tooth should be extracted.

The decision of consultation of physicians was that the man was suffering from a severe attack of neuralgia. In the evening of the second day thereafter, the patient died. Then it was decided to extract the recently filled tooth to see if that had anything to do with the sad result. Immediately the tooth was removed such quantities of matter squirted out of the antrum as to astonish all present. It was found that a quantity of amalgam had been pressed up into the cavity, and that it contained so much free mercury that the "paste" had not yet hardened.—*Items of Interest.*

### ALVEOLA ABSCESS.

By Prof. J. Taft, in Mississippi Valley Association.

"Sometimes an abscess heals spontaneously. If caused by *debris* of the pulp and the latter be removed, the parts will be restored. Usually the cause or causes, are removed; but if they cannot be removed, the trouble then keeps up. There may be a deposit at the end of the root, which cannot be reached. Caustic treatment is not always required. Clean away the irritant and do not over-treat. Should the periosteum be diseased, it may keep up the suppuration. Carbolic acid not only acts as a stimulant, but coagulates the albumen, thus filling up the abscess cavity. Generally it is so used that harm results. Gentle stimulus is the true treatment, after *debris* is removed. Should a fistulous opening exist, fill the root and treat through the fistula."

### EXTRACTION OF TEETH DURING PREGNANCY.

Some writer in the *Cosmos*, signing himself "H. H. B.," says, "The experience of my tutor for thirty-five years, and my own for twelve years has (have ?) taught me never to extract a tooth for a woman during pregnancy for fear of bad results. In lieu I give sedatives to such patients with topical treatment of aching teeth."

And thus, H. H. B. has done as much mischief as he well could with so few words. Thirty-five, and twelve years look formidable. A man ought to learn very much in periods so long. But how has he been taught? How many aching teeth has he extracted for pregnant women? How many his preceptor? How often has such extractions been followed by "bad results? And how bad were these results? And in what did their badness consist?"

Ever since 1844 we have done more than our share of extracting, and we have never, either as physician or dentist, hesitated to extract aching teeth for pregnant women, whenever the toothache could not be otherwise controlled. Any other practice is cruel and dangerous. Five minutes endurance of toothache is more likely to cause miscarriage than is extraction, provided the operation is performed by one fit to extract teeth. Often, and at almost all stages of gestation, have we extracted aching teeth to prevent miscarriage; and invariably with the most satisfactory results. A young married woman suffered with toothache almost the entire period of gestation. The offending tooth was beyond redemption; but she had been taught, as had her physician, that it was necessary for her to bear the pain. When it was supposed that labor had begun, we were sent for to extract, and the offending member was quickly removed. In a very brief period the uterine pains abated, and her confinement did not occur for several days. This is not a solitary case. We have had others nearly similar. The trials of maternity are sufficiently fearful, without the addition of unnecessary torture from toothache.

But when a woman in this trying condition is to submit to the operation of extraction, prudence should be exercised by the operator. All undue excitement should be avoided. The patient should be soothed and pacified. Kindness and firmness combined in the operator will give her confidence; and thus the nervous shock will be greatly mitigated, if not banished. Of course, it is not a good practice to extract when the pain is otherwise readily controllable. A woman engaged in the great work of adding a body, soul, and spirit to the catalogue of the human race, is to be disturbed as little as is practicable; and it is because extraction is a lighter tax on her vital force than is prolonged toothache, that this practice is here recommended. To subject a woman to three, six, or nine months torture which is altogether unnecessary, is malpractice, in its most barbarous aspect. We advise H. H. B. to revise his experience.—*Ohio State Journal of Dental Science.*

### DIGESTION OF TOOTH-PULP.

When a portion of tooth-pulp is dead, or dying, while subjacent parts are alive, with normal circulation, it is very important to get rid of the dead or dying portion, without undue irritation of the parts beneath. Some have claimed that they can cut away the dead surface with a sharp instrument, and yet save alive a good portion of the pulp. The operation must be very delicate; and, in view of present attainments and methods, we think it is quite unnecessary. The pulp is a soft tissue, possessed of a high degree of vitality; and it no more follows that it must be extirpated on account of a sore on its surface, than that a finger must be amputated on account of a felon. The dead tissue must be removed, however, but this is better done by digestion. At the late meeting of the Mississippi Valley Association, Professor J. Taft suggests its removal by pepsin paste, prepared, by mixing to proper consistence, the liquid and powdered pepsin, or by mixing the powder with dilute hydrochloric acid. This is effective. For the same purpose Dr. Van Antwerp suggests the use of the extract of the rind of papaw. This, he says, will "digest the dead portion of a pulp, and not touch the living tissue remaining." Dr. V. is good authority. We are not familiar with this extract, nor with its uses. Is it officinal, and obtainable in the drug stores? Or must the operator prepare it himself? And, if so, how? Will Dr. V. tell us about it in the next number of the JOURNAL? Of course he will. He's too kind to refuse.—*Ohio State Journal of Dental Science.*

[And Dr. Watt will send us a copy?—EDS. INDEPENDENT PRACTITIONER.]

### EXPERT TESTIMONY—A SENSIBLE JUDGE.

The Toledo *Medical Journal* refers to a trial in Toledo in which two physicians had given their testimony as to the facts and were then asked to testify as experts. This they declined doing without compensation. Instead of committing them for contempt, the judge dealt with the case in this way: "This question is one that may be asked, and it is a legal question. But it is calling upon these gentlemen for their opinion, which is the result of money, and study and deep thought for many years, and asking them to do all this for a mere pittance of seventy-five cents a day. It is wrong, there is no question about it. Practically they are right. The law has not provided for this thing. It is a casus omissus; it is a thing which, if the attention of the Legislature had been directed, would have been provided for, because it is just and right. There is no more right in calling upon these medical gentlemen for their years of labor and study and expense, than there would be to call upon a lawyer. Suppose a man has an important matter on which he wants advice, and takes it to one of the lawyers here and says, 'I want your legal opinion;' is there any respectable lawyer who would consent to look after this matter without charging for giving his opinion? Well, now, it is precisely that sort of opinion that our medical men are called upon to give. They are entitled to fair and respectable compensation, and they ought not to be compelled, in my view, to give the result of their observation and thought when it comes from all these years of labor. I therefore will not force that question unless the witness sees fit voluntarily to answer it. In this instance the power of the law might be used, but it would do what is substantially wrong—asking from a man a thing that is valuable and for which there no compensation for."—*Pacific Medical and Surgical Journal.*

HOT WATER COMPRESSES IN TETANUS AND TRISMUS.—Sporer has successfully treated cases of tetanus by merely applying to the nape of the neck and along the spine large pieces of flannel dipped in hot water, of a temperature just bearable to the hand (50–55° C.). —*Allg. Med.-cent. Zeit.*, January 15, 1881.

